# Messages from the Inside. The Dynamic Environment that Favors Intestinal Homeostasis

**DOI:** 10.3389/fimmu.2013.00323

**Published:** 2013-10-09

**Authors:** Rajaraman Eri, Marcello Chieppa

**Affiliations:** ^1^Mucosal Biology, School of Human Life Sciences, University of Tasmania, Launceston, TAS, Australia; ^2^Laboratory of Experimental Immunopathology, IRCCS “De Bellis,” Castellana Grotte, Bari, Italy

**Keywords:** mucosal immunology, intestinal evolution, DCs, intestinal epithelial cells, intestinal inflammation

## Abstract

An organism is defined as “an individual living thing capable of responding to stimuli, growing, reproducing, and maintaining homeostasis.” Early during evolution multicellular organisms explored the advantages of a symbiotic life. Mammals harbor a complex aggregate of microorganisms (called microbiota) that includes bacteria, fungi, and archaea. Some of these bacteria have already defined beneficial roles for the human host that include the ability to break down nutrients that could not otherwise be digested, preventing the growth of harmful species, as well as the ability to produce vitamins or hormones. It is intuitive that along the evolutionary path several mechanisms favored bacteria that provided advantages to the host which, in return, avoided launching an aggressive immunological response against them. The intestinal immunological response does not ignore the lumenal content, on the contrary, immune surveillance is favored by continuous antigen sampling. Some intestinal epithelial cells (ECs) are crucial during the sampling process, others actively participate in the defense mechanism. In essence the epithelium acts as a traffic light, communicating to the inside world whether conditions are safe or dangerous, and thus influencing immunological response. In this review we will discuss the dynamic factors that act on the intestinal ECs and how they directly or indirectly influence immune cells during states of health and disease.

## Evolution of the Digestive System

**Figure 1:**
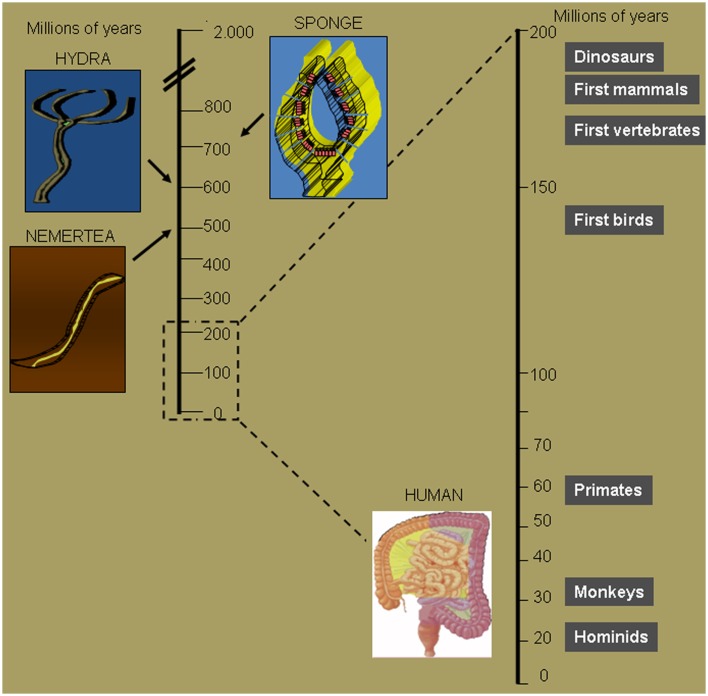
**Crucial steps of intestinal tract evolution**. Seven hundred millions of years ago sponges were the first multicellular organisms to obtain and digest food particles by filtering water. Six hundred millions of years ago Cnidarians evolved a single opening followed by a cavity that serves as a digestive space. The opening served both as the entrance for food and the exit for waste. It was not until about 100 million years later that, with the Nemertea, waste was eliminated through a second opening, thus maximizing food absorption potential. The first mammals evolved 180 million years ago, but the human intestine first appeared about 160 million years later.

Evolution is defined as the change in the inherited characteristics of biological populations over successive generations. Evolutionary processes give rise to diversity at every level of biological organization, including species, individual organisms, and molecules such as DNA and proteins (1). Life on Earth originated 3.7 billion years ago. Given that an organism requires the intake of energy to live, several strategies appeared during evolution to obtain efficient food intake and digestion (Figure 1). Organisms that were more efficient in capturing and digesting nutrients could prevail in the battle for the survival of the species. Sponges are recognized as the first multicellular organism to appear on Earth. These multicellular organisms did not have a specialized digestive tract, every single cell obtained and digested food particles by filtering water (2). An important step in the evolution of the digestive system is represented by the digestive sac. Cnidarians are multicellular organisms that represent a crucial step for the evolution of the gastrointestinal tract as they evolved a single opening followed by a cavity that served as a digestive space where extracellular digestion produces products ultimately distributed to the entire body (3). *Hydra* in particular are elegant multicellular organisms that use their tentacles to introduce food in their gastrovascular cavity (4). Among *Hydra* another crucial evolutionary step took place in the *Hydra viridis*. This common organism belonging to the phylum Cnidaria appeared on Earth 580 million years ago. Its characteristic green color derives from cells of the unicellular alga symbiotically living within the cells of the gastrodermis. Maintenance of normal symbionts within host digestive cells at relatively constant numbers is due to their avoidance of host digestion. Symbionts continued to evolve together with the host organism. In humans a complex digestive tract harbors an aggregate of microorganisms (called microbiota) that includes bacteria, fungi, and archaea. Some of these bacteria have already defined beneficial roles for the human host, which include the ability to break down nutrients that could not otherwise be digested, preventing the growth of harmful species, as well as the ability to produce vitamins or hormones. It is intuitive that along the evolutionary path several mechanisms favored the microbiota that was able to provide advantages to the host which, in return, offered a safe home where food routinely arrived. A sick host will stop providing food, thus negatively selecting for microbiota that damaged the host by favoring inflammatory responses or harmful infections. At the same time the host has the challenging duty of allowing controlled microbiota growth and of reacting against possible treats.

### Intestinal epithelial cells

The intestinal epithelial is a monolayer of cells responsible for the absorption of nutrients taking place through the epithelial cells’ (ECs) luminal side. To maximize this process and obtain the largest surface area the small intestine consist of villi and crypts that tremendously increase the number of ECs. Furthermore the luminal surface of the ECs presents microvilli that further increase the exchange surface area. In humans the overall surface covers approximately 200 m^2^ (5). This strategy perfectly serves the need to increase nutrient absorption, and at the same time exposes the intestine to the largest possible contact area between the body and the external world via the intestinal lumen. More than 160 species of bacteria populate the intestinal lumen (6). Their density increases along the length of the intestine, peaking in the colon where water is reabsorbed and bacteria are packed and expelled as a major component of feces. Since the first phases of gastrointestinal evolution the ECs monolayer was exposed to challenging conditions given the need for it to be simultaneously a physical, chemical, and electrical barrier between the sterile internal environment and the non-sterile external one. This complex luminal micro-environment is called microbiota and includes bacteria, fungi, nematodes, and viruses. The microbiota has the ability to break down nutrients, produce vitamins or hormones, and prevent the growth of harmful species, all factors that represent an advantage to the host (7). Nevertheless the microbiota is not ignored by the host’s defenses, as immune cells and ECs assemble a series of strategies to achieve active vigilance of the luminal content (8–11). All these mechanisms contribute in maintaining a stable state of the internal environment, which is maintained by regulatory processes despite changes that may occur in the external environment, a phenomenon named “homeostasis” in 1929 by W.B. Cannon (12).

### Epithelial cells secrete factors that shape the intestinal lumen content

The gastrointestinal tract has long been considered to contain the largest number of lymphocytes in the human body, and although recent studies have revised this idea, it has been confirmed that a significant percentage – ranging between 5 and 20% of all lymphocytes – resides in the gut (13). A dense net of immune cells underlines the ECs monolayer, with distinct aggregates of lymphoid follicles structured as isolated lymphoid follicles (ILFs) in the colon or Peyer’s Patches in the small intestine. The intestinal lumen is also the site where the greatest amount of immunoglobulin is secreted, indeed IgA producing B cells are largely “instructed” in the PPs. IgA are extremely important to the correct bacterial distribution along the intestine (14) as demonstrated by an elegant experiment using immunocompetent (scid/+) or immunodeficient (scid/scid) mothers. The neonatal intestinal distribution of Segmented Filamentous Bacteria (SFB) was related to the passively acquired maternal secretory IgA (sIgA) in the milk. Milk lacking IgA favored the abnormal SFB colonization of the ileum (15). IgA induction is extremely limited under germ free conditions, but can be rapidly established following intestinal colonization (16). A proliferation-inducing ligand (APRIL) is a key factor for the class switch recombination to IgA, and its production requires TLRs engagement by intestinal ECs (17). sIgA are transported across the ECs into the lumen by the polymeric immunoglobulin receptor (pIgR). As sIgA represent the first line of defense preventing an unneeded pro-inflammatory response as a consequence of the adherence of bacteria to the mucus layer, the induction of pIgR has to be constantly sustained (18–20). Commensal bacteria represent at the same time the stimulus for the coordinated signals that enhance sIgA production and pIgR expression. Intestinal bacteria promote ECs Thymic Stromal Lymphopoietin (TSLP) production (21). TSLP is an IL-7 related cytokine originally isolated from a mice thymic stromal cell line (22). TSLP signaling is mediated by TSLP binding to the IL-7Rα and TSLPR. Experiments that used different approaches, including the TSLPR-deficient mice, were crucial in demonstrating the importance of the TSLP–TSLP axis in the production of APRIL (23), Th2, and Foxp3+ regulatory T cells (T_regs_) induction (8–11, 24, 25) as well as Th1, Th17 inhibition (26, 27).

### Mucosal layer mediated protection

Epithelial cells are involved in the production of other key players in correct intestinal homeostasis. Among them the mucus layer and the antimicrobial protein (AMP) contribute significantly. The mucus layer is produced by the goblet cells and is fundamental for the protection of the gastrointestinal tract. Its anatomical distribution is consistent with the need to protect the epithelial monolayer and create a disconnection between the body and the luminal content. Indeed the small intestine does not present a well defined mucus layer, as opposed to what happens in the colon and in the stomach (28). The colonic mucus layer is organized in an insoluble inner layer that is relatively sterile, protecting the ECs from bacterial encounter, and a loose outer layer that is well colonized by commensal bacteria (29). MUC2 is the major component of the mucus layer in the small and large intestine and mutations that involve MUC2 are related with chronic intestinal inflammation as a result of uncontrolled ECs exposure to the commensal bacteria (30, 31). Another important role of the mucus layer is to concentrate the epithelial AMPs near the epithelial surface. AMPs production is another fundamental mechanism for commensal control and selection. Enterocytes are the major producer of these proteins (32), but immune cells can also efficiently contribute (33). Diverse AMPs are produced in the small or large intestine and even among the same anatomical compartment, while different cells produce different AMPs. Paneth cells located at the base of the intestinal crypts express α-defensins (34) and RNase (35), while enterocytes produce C-type lectins in the small intestine (36) and β-defensins in the colon (35). Together with the antimicrobial effects, these proteins affect the intestinal immune response and in particular contribute to shape the inflammatory response mediated by the intestinal dendritic cells (DCs) underlying the ECs monolayer (37).

### Intestinal dendritic cells

Dendritic cells are defined as the most potent antigen presenting cells, able to capture, process, and present antigens to initiate the adaptive immune response (38). DCs are distributed all the way through the gut in the lamina propria (LP), gut associated lymphoid tissue (GALT), and in discrete lymphoid aggregates (the latter are generally present in the colon). DCs activate a series of maturational processes in response to microbial antigens exposure that are involved in the innate antimicrobial and inflammatory responses. Furthermore DCs maturation activates T and B cells, initiating the adaptive immune responses (39). Microbial antigens can be detected by the DCs following traumatic events that perturb the natural sterile habitat of the human body. This is not true in tissues like the intestine or the skin where microbes are not only tolerated but even welcome. As aforementioned, the microbiota is an important player for food digestion, vitamin production, and even defense against potential pathogens. The symbiotic coexistence between bacteria and host preexists the development of the immune system, it is therefore not surprising that the immune system evolved mechanisms to avoid potentially dangerous inflammatory responses in these compartments. Intestinal DCs are pivotal for sustaining immune tolerance toward oral antigens. Indeed, DCs promote differentiation, expansion, and maintenance of T_reg_ (40) and the induction of IgA producing B cells against commensal bacteria. The adaptive immune responses initiate in the mesenteric lymph node (MLN) where DCs migrate from the intestine. Worbs et al. demonstrated that genetic defects that alter CCR7 mediated trafficking profoundly affect the induction of tolerance to oral antigens (41).

### Intestinal DCs subtypes

Different DCs subtypes coexist in the intestinal LP. Iwasaki et al. first defined intestinal DCs based on the expression of markers such as CD8α, CD11b, and CD11c (42, 43). More recently the expression of αEβ7 (CD103) has been associated with the DCs subset migrating to the MLN and promoting the tolerogenic response. Indeed CD103+ DC are characterized by high levels of retinoic acid (RA) synthesizing enzyme (RALDH), which is crucial for the T_reg_ inducing capacity (44). CX3CR1 is another crucial marker that has been used to discriminate intestinal DCs subpopulation. CX3CR1^+^ DCs do not express CD103 and, in marked contrast with the CD11c^+^CD103^+^ DCs, do not migrate to the MLN and are poor T_reg_ inducers (45, 46). In summary MHCII^+^, CD11b^+^, CD11c^+^, CD103^+^,CX3CR1^−^ cells are migrating DCs able to promote T_reg_ conversion, imprint gut-homing properties and induce IgA switch, while MHCII^+^, CD11b^+^, CD11c^+^, CD103^−^,CX3CR1^+^ are TNFα producers involved in Th17 induction. Starting from the observation that CX3CR1^+^ DCs are not the MLN migrating DCs, a recent work by McDole et al. (47), focused on CD103^+^ DCs antigen uptake. This elegant study, which used minimally disruptive *in vivo* imaging, suggests that small intestine goblet cells deliver soluble luminal antigens to the underlying CD103^+^ DCs. Several studies described the DCs ability to extend processes between ECs, both along the small intestine (46, 48, 49), in the PPs (50), and the trachea (51). This ability has been related with the need to uptake luminal antigens to be presented in the MLN even in the absence of inflammation. In this regard, Farache et al. were able to distinguish the antigen uptake ability of two different DCs populations. Using a CD11c-YFP × CX3CR1^+/gfp^ mouse model, they described a population of CD103^+^ GFP^−^ YFP^int^ DCs that are able to internalize non-invasive *Salmonella* by sending dendrites between ECs. At the same time these cells were not as efficient in the uptake of soluble OVA injected into the intestinal lumen, which was mostly internalized by the CX3CR1^+^ macrophage population (46). In addition to the aforementioned uptake mechanisms, an alternative sampling/capturing mechanism has been recently studied. Arques et al. observed (CD11c^+^, CX3CR1^+^, MHCII^+^, CD11b^−^, CD8a^−^) DCs trans-epithelial migration in the small intestine of *Salmonella* treated mice (52). The authors suggest that DCs migration in response to TLR5 engagement represents a strategy to prevent or limit the number of pathogens that can penetrate the intestinal epithelium. These hypotheses deserve to be further investigated but represent fascinating mechanisms for the mucosal immune response. At the same time, it is important to better understand the molecular mechanisms that regulate DCs luminal sampling. Different approaches demonstrated that DCs intralumenal sampling is a chemokine related phenomenon (52), but the key factor has still to be identified. It is important to underline that trans-epithelial dendrites have been clearly observed *in vitro* and *in vivo*, but considerable differences between studies need to be carefully considered, including those that argue against the relevance of such a mechanism *in vivo* (46, 47, 53, 54). The *in vivo* observation of the intestinal lumen requires an invasive procedure that may create acute inflammation and alter the epithelial barrier permeability, eventually inducing apoptosis. Variability between studies may be related to differences in the animal facility condition, starvation periods, or by the procedures required to obtain clean observation areas. Altogether it is clear that intestinal homeostasis is the result of dynamic processes based on vigilant tolerance mediated by antigen presentation in the absence of inflammation. DCs migrating in the MLNs provide the intelligence that decides the outcome of the adaptive response, for this reason so much effort has been devoted to define the intestinal derived factors able to educate the intestinal DCs.

### Epithelial derived immune factors

**Figure 2:**
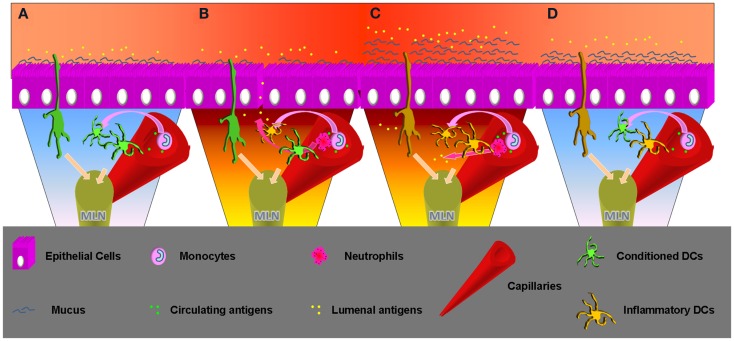
**Time progression, from homeostasis to inflammation and inflammation remission**. **(A)** Homeostasis: lumenal and epithelial derived factors imprint an inflammatory impaired phenotype to the intestinal resident DCs. These can sample antigens from the lumen but also from the capillary, but migrating to the MLNs will not produce inflammatory cytokines and will not activate an aggressive adaptive response. Incoming DCs progenitor will enter a favorable environment becoming inflammatory impaired. Sampling circulating antigens these DCs may be crucial to sustain tolerance toward self. **(B)** Inflammatory insult: epithelial barrier loss or invasive bacteria can change the intestinal milieu, which will lose the ability to condition incoming DCs progenitors. These will possibly encounter lumenal antigens and migrate to the MLN to begin a Th1/Th17 adaptive response. Neutrophils will be recruited as well by the epithelial produced IL-8/KC. Production of this chemokine appears to be mediated by TLR5 engagement that happens in cases of infection. Previously conditioned DCs will not be able to produce an inflammatory response, but migrating to the MLN their effect will be stochastically surmounted by the freshly recruited DCs if the inflammation is prolonged. **(C)** Inflammation: incoming inflammatory cells release TNFα that promotes mucus production by goblet cells. A thicker mucus layer better protects from lumenal antigen exposure, important to create the conditions to interrupt the pro-inflammatory cascade. At the same time neutrophils and macrophages clean-up the lamina propria. Incoming DCs progenitors retain the possibility of becoming inflammatory, but the chances decrease in relation with the successful resolution of the infection. Intestinal DCs that sample circulating antigens in pro-inflammatory conditions inside the capillaries may erroneously induce inflammatory responses toward self-antigens. This may enlighten new aspects related with systemic inflammatory responses observed in patients affected by chronic intestinal inflammation. **(D)** Inflammatory remission: the epithelial barrier is back to normal and epithelial cells are exposed to sustainable amount of antigens. The ECs cytokine cocktail favors DCs polarization to the conventional intestinal phenotype. DCs migrating to the MLN will produce increasing amounts of anti-inflammatory cytokines and correct intestinal homeostasis is finally completed.

The intestinal epithelium evolved to become the largest surface of the body in contact with the external world. The intestinal lumen represents an extremely challenging environment where the need to protect the body from external treats has to coexist with the necessity to permit nutrient absorption. For this reason the largest amount of immune cells resides in close contact with the intestinal ECs. Among them DCs continuously receive signals from the ECs to verify if microbes and ECs are interacting properly. ECs are indeed able to recognize pathogens through a variety of pattern recognition receptors (PRR), including several TLRs expressed on their luminal or basolateral surface (55– 57). ECs response to TLRs ligand discriminate TLRs from the luminal or basolateral side. Lumenal TLRs represent constitutive conditions related with the presence of the intestinal microbiota; ECs respond to TLRs engagement by NFkB translocation into the nucleus that leads to the production of chemokines, cytokines (58), and regulate epithelial integrity (59). Basolateral TLRs engagement is more likely related with bacterial invasion or epithelial monolayer discontinuity that results in different types of ECs activation and consequently a pro-inflammatory response (Figure 2). In case of bacterial invasion DCs underlying the ECs monolayer should not be able to respond in an inflammatory way. In fact, DCs conditioned by ECs supernatant lose the ability to produce IL-12 while producing large amounts of IL-10 even following *Salmonella* exposure (11, 60). Efficient inflammatory response needs to be activated by incoming monocytes that differentiate between DCs in a pro-inflammatory environment (61). Resident DCs conditioning is obtained by ECs release of soluble factors – including TSLP (62) and TGF-β (31) – that appear to synergize *in vitro* (63). Intestinal DCs also appear to be able to extend processes inside the capillaries of the LP to sample circulating antigens (64). This important observation may shed new light on the connection between inflammatory events that occur in the intestinal compartment and systemic loss of tolerance against self-antigens. More immunomodulating factors may arrive from the intestinal lumen. On this question Agace and Persson recently contributed to the understanding of the role of luminal content in imprinting the unique phenotypic and functional characteristic of intestinal DCs.

### Lumenal products conditioning immune cells

Food derived factors and the entire microbiota can influence intestinal homeostasis affecting the epithelium or immune cells directly. Vitamins, for example, act directly on the immune system. Vitamin A was recently linked to intestinal immune response as vitamin A metabolite RA is crucial in imprinting gut-homing properties on T and B cells (65). Vitamin A is acquired through diet but its active form requires the action of RALDSs to obtain *all-trans-*RA. Together with the aforementioned gut-homing properties, RA promotes T_reg_ and inhibits Th17 differentiation (66). Another RA important feature is the ability to promote B cell class switch imprinting IgA secretion abilities fundamental for correct gut homeostasis and intestinal flora control. Vitamin D, E, and C can act as antioxidants able to modulate immune response (67, 68). Polyphenols are food derived antioxidants also capable of fine-tuning immune response by modulating the maturation of the DCs as shown by the ability of curcumin and resveratrol to suppress inflammatory cytokine secretion through *in vitro* cultured DCs exposed to LPS (69). Notably, an interesting study by Smith et al. describes the ability of microbial metabolites short-chain fatty acids, to directly enhance colonic T_reg_ frequency via GPCR43 expressed by the immune cells (70). Overall, the idea of nutraceuticals (products that have both nutritional and pharmaceutical qualities), a term coined by Stephen DeFelice in 1989 (71), is becoming ever more interesting as we acquire information on how to direct immune response toward tolerance or inflammation, as appropriate. It’s not surprising that the major apparatus involved in food derived immunomodulation is the one that is the most exposed to food derived products. An appreciation of the systemic relevance of gut imprinted tolerogenic response will teach us how to modulate inflammation and eventually prevent and cure chronic inflammatory syndromes.

## Conclusion

In conclusion, intestinal homeostasis is maintained in a dynamic equilibrium by balancing the contribution of different players. Nutritional intake has been the driving force shaping the system and the microbiota, and the body evolved consequently. Many aspects of mucosal immune response have been discovered during the last few decades, but it appears evident that we are still far from a full understanding of the complexity of the system. Oversimplification obtained by *in vitro* studies or extreme conditions recreated in various mice models are needed to dissect and understand key elements of the system. Nonetheless the complete pattern is still not quite clear, while the rate of intestinal inflammatory disorders is increasing worldwide, with the consequent urgent need for new and more efficient treatments.

## Conflict of Interest Statement

The authors declare that the research was conducted in the absence of any commercial or financial relationships that could be construed as a potential conflict of interest.
